# Biomechanical effects of hybrid constructions in the treatment of noncontinuous cervical spondylopathy: a finite element analysis

**DOI:** 10.1186/s13018-023-03537-7

**Published:** 2023-01-20

**Authors:** Xiangyao Sun, Qingming Zhang, Li Cao, Juyong Wang, Jiang Huang, Yuqi Liu, Yang Zhang, Zelong Song, Wei Tang, Yunqiang Chen, Siyuan Sun, Shibao Lu

**Affiliations:** 1 Department of Orthopaedics, Xuanwu Hospital Capital Medical University, Beijing, 100053 China; 2 National Clinical Research Center for Geriatric Diseases, Beijing, 100053 China; 3 Beijing Glitzern Technology Co., Ltd, Beijing, 100077 China; 4 Department of Emergency, Xuanwu Hospital, Capital Medical University, Beijing, 100053 China; 5 Department of Orthopaedics, The Seventh Medical Center of PLA General Hospital, Beijing, 100191 China; 6 Nankai University School of Medicine, Nankai University, Tianjin, 300071 China; 7 Department of Orthopaedics, The PLA General Hospital, Beijing, 100000 China; 8 Department of Interdisciplinary, Life Science, Purdue University, West Lafayette, IN 47907 USA

**Keywords:** Hybrid construction, Total disc replacement, Anterior cervical discectomy and fusion, Noncontinuous cervical spondylopathy, Biomechanics

## Abstract

**Background:**

Hybrid construction (HC) may be an ideal surgical strategy than noncontinuous total disc replacement (TDR) and noncontinuous anterior cervical discectomy and fusion (ACDF) in the treatment of noncontinuous cervical spondylopathy. However, there is still no consensus on the segmental selection for ACDF or TDR in HC. The study aims to analyse the effects of different segment selection of TDR and ACDF on cervical biomechanical characteristics after HC surgery.

**Methods:**

Twelve FEMs of C2–C7 were constructed based on CT images of 12 mild cervical spondylopathy volunteers. Two kinds of HC were introduced in our study: Fusion-arthroplasty group (Group 1), upper-level (C3/4) ACDF, and lower-level TDR (C5/6); Arthroplasty-fusion group (Group 2), upper-level (C3/4) TDR and lower-level ACDF (C5/6). The follow-load technique was simulated by applying an axial initial load of 73.6 N through the motion centre of FEM. A bending moment of 1.0 Nm was applied to the centre of C2 in all FEMs. Statistical analysis was carried out by SPSS 26.0. The significance threshold was 5% (*P* < 0.05).

**Results:**

In the comparison of ROMs between Group 1 and Group 2, the ROM in extension (*P* = 0.016), and lateral bending (*P* = 0.038) of C4/5 were significantly higher in Group 1 group. The average intervertebral disc pressures at C2/3 in all directions were significantly higher in Group 1 than those in Group 2 (*P* < 0.005). The average contact forces in facet joints of C2/3 (*P* = 0.007) were significantly more than that in Group 2; however, the average contact forces in facet joints of C6/7 (*P* < 0.001) in Group 1 group were significantly less than that in Group 2.

**Conclusions:**

Arthroplasty-fusion is preferred for intervertebral disc degeneration in adjacent upper segments. Fusion-arthroplasty is preferred for patients with lower intervertebral disc degeneration or lower posterior column degeneration.

*Trial registration*: This research was registered in Chinese Clinical Trial Registry (ChiCTR1900020513).

## Background

Cervical spondylopathy is a commonly seen degenerative spinal disease, which can decrease the patient’s motor function and quality of life [1]. Degenerative changes of cervical discs and ligamentous hypertrophy can attribute to chronic spinal cord compression and then lead to the progression of cervical spondylopathy [2]. Noncontinuous cervical spondylopathy is a special kind of multilevel cervical spondylopathy, the condition of which is the normal intermediate segment located between two noncontiguous degenerative segments [3].

Anterior cervical discectomy and fusion (ACDF) is the most widely used surgical method in the treatment of cervical spondylopathy, which can decompress nervous structures and achieve cervical realignment [4]. However, ACDF may increase the stress of intervertebral discs and endplates as well as facet joints overloading, which can cause adjacent segment degeneration (ASD) and cervical instability [5]. Previous studies ameliorated this procedure with skip-level ACDF, which included single segment titanium plates and self-locking cages or zero-p; it is reported that this method could achieve satisfactory clinical outcomes [6–8]. Although the intermediate segment is not fused in this procedure, it will stand with more additive stress from fused segments on the upper and lower sides, as well as compensative hypermobility for fused segments, which can accelerate intervertebral disc degeneration [3].

Cervical disc prosthesis, which is called cervical total disc replacement (TDR), can maintain physiological cervical movement instead of intervertebral fusion; this can decrease kinematic overloading at upper and lower adjacent segments [1]. TDR is usually carried out at the intervertebral level without instability or facet joint degeneration [9]. In addition, the facet joint can be overloaded at the TDR level, while the force transmission decreases at the adjacent levels [10, 11].

Hybrid construction (HC) combines TDR and ACDF together [9]. Previous studies yield that HC could provide ideal clinical outcomes and radiologic parameters; in addition, HC would not significantly change the total range of motion (ROM) or the ROM of adjacent segments [9, 12, 13]. HC may be an ideal surgical strategy than noncontinuous TDR and noncontinuous ACDF in the treatment of noncontinuous cervical spondylopathy. There is still no consensus on the segmental selection for the usage of ACDF or TDR in HC. Previous finite element analysis (FEA) simplified the vertebral distraction during the insertion of an intervertebral implant in the models, which could not simulate the real biomechanical environment [14]. In addition, most previous biomechanical studies just used the data of one case to draw a conclusion without statistical support, which could affect the accuracy of its conclusions [14–16].

To provide more convincing evidence for the implementation of HC, we performed FEA on the cervical biomechanical characteristics of noncontinuous cervical spondylopathy patients. The data obtained from FEA were statistically analysed. We hope that this study can analyse the effects of different segment selection of TDR and ACDF on cervical biomechanical characteristics after HC surgery.

## Methods

### The geometry models

In this study, FEA technology is based on computed tomography (CT) images (SOMATOM Definition AS + , Siemens, Germany, a thickness of 0.75 mm and an interval of 0.69 mm) of the cervical spine (C2–C7) to build real clinical models, which is effective in construct finite element models (FEMs) from radiologic information [17]. This modelling technique can help surgeons make an optimal surgical strategy by predicting potential risks with satisfactory precision and effectiveness. Twelve mild cervical spondylopathy volunteers were included in this study (six male and six females), whose DICOM format CT images were fully achieved. These radiographic images were used to reconstruct geometric models of C2–C7 cervical vertebrae in Mimics 17.0 (Materialize Inc, Leuven, Belgium). The geometric models were output as STL files and then imported into Geomagic Studio 12.0 (3D System Corporation, Rock Hill, SC, USA) to adjust these models into physical structures.

**Fig. 1:**
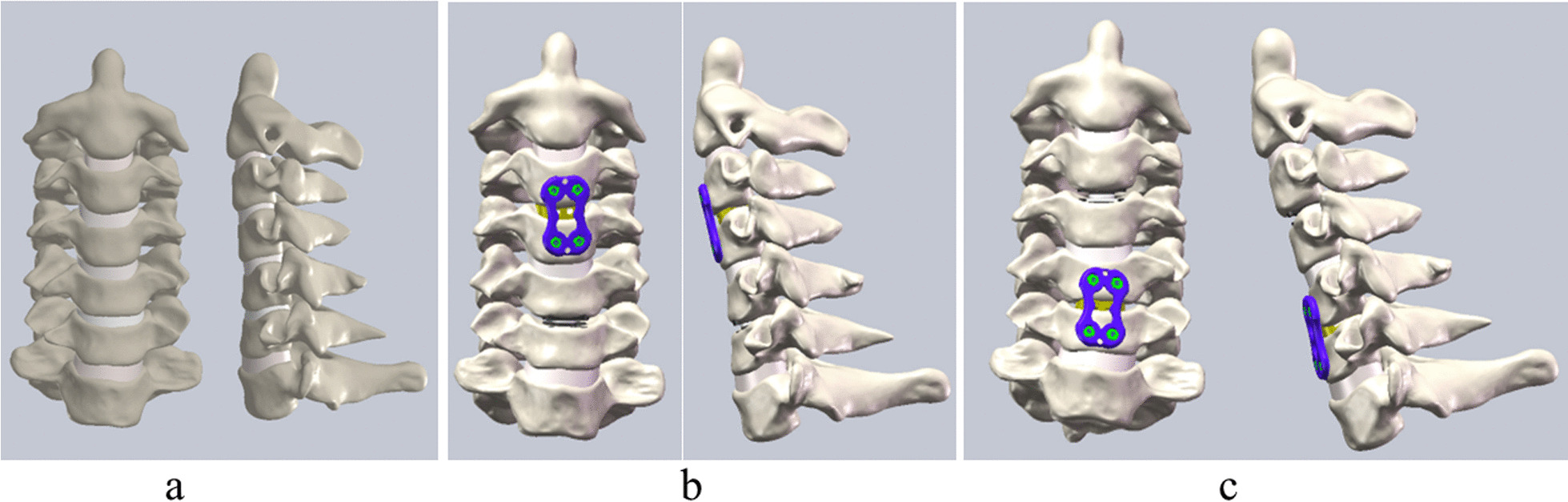
FEMs of intact group (**a**), Fusion-Arthroplasty group (Group 1) (**b**), and Arthroplasty-fusion group (Group 2) (**c**)

The implants analysed in this study included NuVasive® Helix ACP (NuVasive, Inc. San Diego, CA, USA), CoRoent® Contour (NuVasive, Inc., San Diego, CA, USA) and Prodisc-C (Synthes, Inc., West Chester, PA, USA). The basic information of implants is shown in Table 1. Solidworks 2016 (Dassault Systèmes, MA, USA) was used to build the FEMs of these devices and assemble them with cervical vertebra models. HC is different from hybrid decompression. The utility of cervical discectomy and corpectomy is called hybrid decompression [18–20]. Therefore, we should not confuse these two concepts. Considering intermediate C4/5 got the most attention in the previous studies on noncontinuous cervical spondylopathy, C3/4 and C5/6 were selected to perform HC in our study [3, 14, 17]. Two kinds of HC were introduced in our study: Fusion-Arthroplasty group (Group 1), upper-level (C3/4) ACDF and lower-level TDR (C5/6); Arthroplasty-Fusion group (Group 2), upper-level (C3/4) TDR and lower-level ACDF (C5/6). All the models were used to perform these different HC strategies in the software mentioned above. In ACDF, NuVasive® Helix ACP and CoRoent® Contour were implanted at intervertebral level after removing relevant intervertebral disc, anterior longitudinal ligament (ALL), and posterior longitudinal ligament (PLL). In TDR, we implanted Prodisc-C at the corresponding level after the resection of relevant intervertebral junctional structures (Fig. 1). In practice, the posterior longitudinal ligament should be removed from the implanted segment to achieve adequate decompression of the neural structure. The models in this study simulated the actual surgical procedure; therefore, they required the removal of the posterior longitudinal ligaments at the implanted segments.

**Table 1:** Basic information of devices

Devices	Manufacturers	General view	Outline dimensions
CoRoent® Contour	NuVasive, Inc., San Diego, California, USA		17 mm long, 14 mm wide, 6 mm high, 7° lordotic
NuVasive® Helix ACP	NuVasive, Inc. San Diego, California, USA		Plate: 16 mm long, 24 mm wide and 2.4 mm thick Screws: diameter of 4.5 mm, 14 mm long
Synthes® Prodisc-C	Synthes, Inc., WestChester, PA		16 mm long, 15 mm wide, 6 mm high

Hypermesh 12.0 (Altair, Troy, MI, USA) was used to construct high-quality meshes of these models. Material properties, experimental conditions, and FEA were then set and carried out in ABAQUS 6.13 (Dassault Systems Corporation, MA, USA).

### Material properties

Material properties and element types are shown in Table 2 [15, 17, 21–23]. In these FEMs, the thickness of cortical shell and endplates was set as 0.4 mm [24]. As to the facet joint, the articular surface was covered with cartilage, which was assigned with nonlinear surface-to-surface contact; the thickness of cartilage and the gap were 0.5 mm [14]. The structure of the intervertebral disc is as follows: Nucleus occupied about 40% volume of the intervertebral discs, and the annulus was around 60%; annulus fibres accounted for about 19% of the annulus fibrosus volume; the inclination of the annulus fibrosus to the transverse plane was 15° to 30°; a tie connection was defined between the parts of intervertebral discs and endplates [3, 14]. In addition, six groups of ligaments included ALL, PLL, ligament flavum (LF), interspinous ligament (IL), supraspinous ligament (SL), and capsular ligament (CL); all of them were inserted into the model [15, 25].

**Table 2:** Material parameters of cervical components and implants

Material	Elastic modulus (MPa)	Poisson ratio	Element type	Cross-sectional area (mm^2^)
*Vertebra*				
Cortical bone	12,000	0.3	C3D4	–
Cancellous bone	100	0.2	C3D4	–
Endplate	500	0.4	S3	–
Cartilage	10	0.4	C3D4	–
Posterior structure	3500	0.25	C3D4	–
*Intervertebral disc*				
Annulus fibre	450	0.45	T3D2	–
Annulus ground Substance	Hyperelasitc Mooney-Rivlin		C3D6	–
	C10 = 0.56			
	C01 = 0.14			
Nucleus pulposus	Hyperelasitc Mooney-Rivlin		C3D6	–
	C10 = 0.56			
	C01 = 0.14			
*Ligaments*				
ALL	30	0.4	T3D2	6.1
PLL	20	0.4	T3D2	5.4
LF	10	0.4	T3D2	50.1
IL	10	0.4	T3D2	13.1
SL	10	0.4	T3D2	13.1
CL	10	0.4	T3D2	46.6
*NuVasive® Helix ACP*				
Ti6Al4V	114.000	0.35	C3D4	–
*CoRoent® Contour*				
PEEK	3400	0.4	C3D4	–
*Synthes® Prodisc-C*				
End plates (cobalt chromium)	210,000	0.3	C3D4	–
Core (polyethylene)	800	0.3	C3D4	–

*ALL* anterior longitudinal ligament, *PLL* posterior longitudinal ligament, *LF* ligament flavum, *IL* interspinous ligament, *SL* supraspinous ligament, *CL* capsular ligament, *PEEK* polyether-ether-ketone, *C3D4* tetrahedron, *S3* shell, *T3D2* truss, tension only, *C3D6* triangular prism

### Experimental condition

The interfaces between natural structures of the cervical spine were assigned with a tie constraint; the contact of facet joints was set to a friction sliding; the C7 bottom surface was set to completely constrained in all degrees of freedom [24]. The simulation of screw-vertebra interface and screw-implant interface was set to a tie constraint; the interface between the cancellous bone graft and CoRoent® Contour was assigned with a frictionless contact; end plates and nucleus of ProDisc-C were set to have full surface contact with a tie constraint [17, 21]. The follow-load technique was simulated by applying an axial initial load of 73.6 N through the motion centre of FEM, which was equivalent to muscle forces and head weight; the motion of the entire C2–C7, including flexion, extension, lateral bending, and axial rotation under the bending moment of 1.0 Nm was applied to the centre of C2 in all FEMs to simulate flexion, extension, lateral bending and axial rotation of entire C2–C7 [10]. The ROM of each intervertebral level was achieved and compared with the published data to validate the reliability of our FEMs [10, 17, 26–28]. This study was concerned with the analysis of assemblies with connection joints or contact conditions. The traditional manual H-element convergence method was used in this study. In addition, the second-order mass element level default element size was used in this study. The initial mesh control was used in this study to ensure that the geometry was consistent with the undeformed. Finally, this study used a local grid to control the desired location to achieve convergence. All FEMs in this study were treated as above.

### Statistical analysis

Statistical analysis was carried out using Statistical Package for the Social Sciences version 26.0 software (SPSS, Inc, Chicago, IL). Mean ± standard deviation (SD) was used to report continuous variables. Normality was tested via the Shapiro–Wilk test. Wilcoxon rank-sum test was used to analyse variables that were not normally distributed. Normally distributed variables were analysed by Student’s *t* test. The significance threshold was 5% (*P* < 0.05).

## Results

### ROM at different levels

**Fig. 2:**
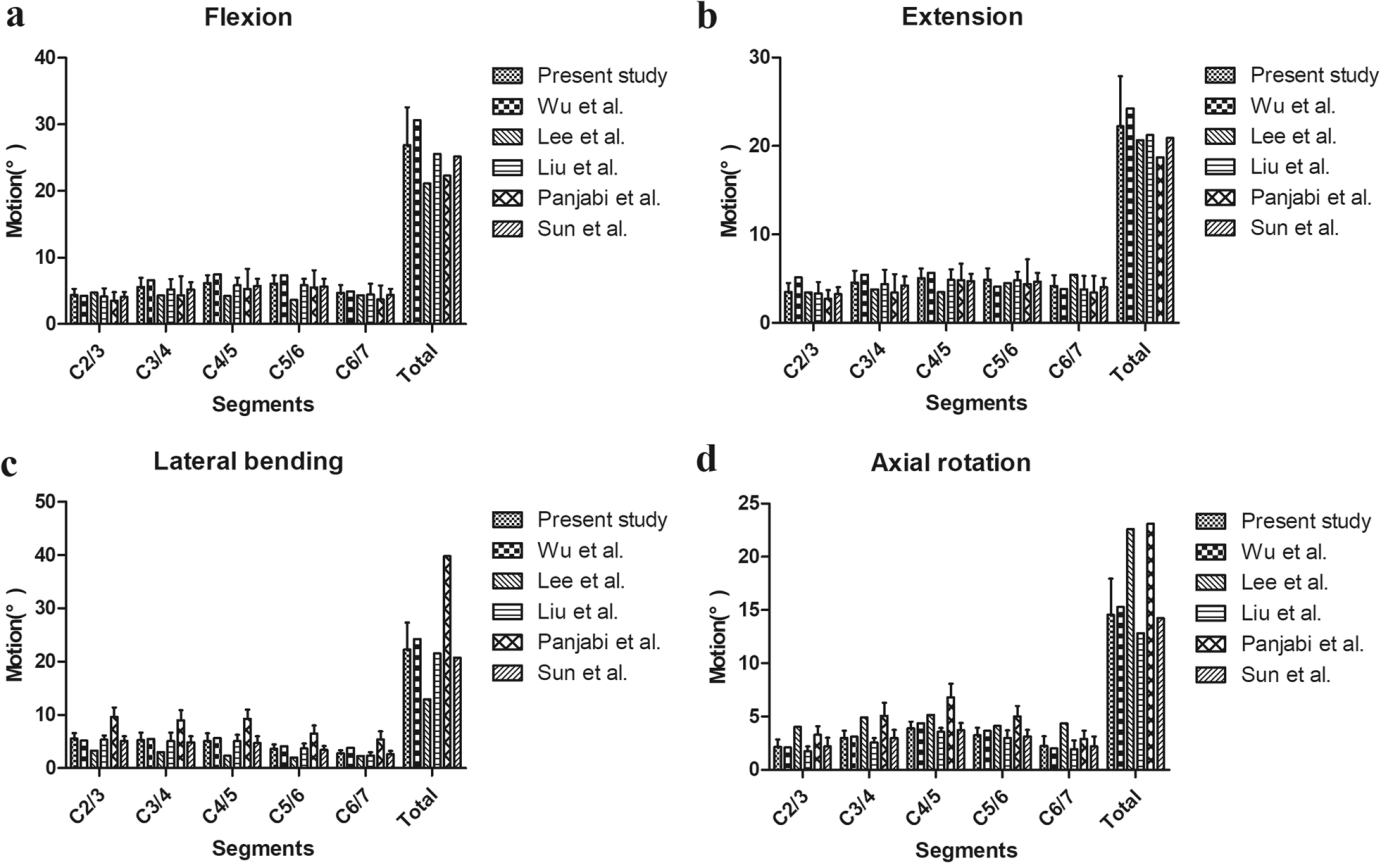
FEMs are validated by previous studies via the comparison of ROMs

The predicted ROM of C2-C7 FEMs was compared with the published data from previous studies (Fig. 2) [10, 17, 26–28]. The average ROMs of intact FEM from C2/3 to C6/7 were in good agreement with previous studies. A comparison of ROMs at different intervertebral levels between the intact group, Group 1, and Group 2 is shown in Table 3. The results showed that the ROMs in lateral bending at the arthroplasty segments were significantly more than that of the corresponding segment under physiological conditions (*P* < 0.05). In the comparison of ROMs between Group 1 and Group 2, the usage of arthroplasty at C5/6 could significantly increase the ROMs of the distal adjacent segment in extension (*P* = 0.015); in addition, the implant of arthroplasty at C3/4 could significantly increase the ROMs of the proximal adjacent segment in lateral bending (*P* = 0.039). The difference in ROMs between implant segments was related to the biomechanical characteristics of implants. No significant difference was found in total ROM between Group 1 and Group 2. This suggested that both treatment strategies had similar effects on the overall ROMs of the cervical spines.

**Table 3:** Comparison of ROMs at different intervertebral levels

Motion (°)	Segments	Models			*P* values		
		Intact	Group 1	Group 2	Intact versus Group 1	Intact versus Group 2	Group 1 versus Group 2
Flexion							
	C2/3	4.36 ± 0.96	4.23 ± 0.90	3.55 ± 0.75	0.423	0.041	0.069
	C3/4	5.61 ± 1.34	0.46 ± 0.10	6.16 ± 1.31	< 0.001*	0.342	< 0.001*
	C4/5	6.14 ± 1.23	5.54 ± 1.18	5.62 ± 1.20	0.132	0.325	0.882
	C5/6	6.12 ± 1.22	3.31 ± 0.28	1.23 ± 0.26	< 0.001*	< 0.001*	< 0.001*
	C6/7	4.68 ± 1.24	4.44 ± 0.94	4.13 ± 0.88	0.345	0.238	0.429
	Total	26.91 ± 5.71	17.99 ± 3.39	20.69 ± 4.39	< 0.001*	0.009*	0.121
Extension							
	C2/3	3.48 ± 1.04	3.05 ± 0.78	2.45 ± 0.62	0.154	0.010	0.057
	C3/4	4.57 ± 1.35	0.52 ± 0.13	5.05 ± 1.29	< 0.001*	0.401	< 0.001*
	C4/5	5.07 ± 1.07	5.66 ± 1.45	4.24 ± 1.08	0.634	0.082	0.016
	C5/6	4.89 ± 1.31	4.62 ± 1.18	0.57 ± 0.15	0.357	< 0.001*	< 0.001*
	C6/7	4.20 ± 1.21	4.75 ± 1.21	3.55 ± 0.91	0.593	0.167	0.015*
	Total	22.22 ± 5.67	18.60 ± 4.75	15.86 ± 4.05	0.119	0.006*	0.159
Lateral bending							
	C2/3	5.57 ± 1.02	3.25 ± 0.73	4.02 ± 0.91	< 0.001*	0.001*	0.039*
	C3/4	5.25 ± 1.42	0.63 ± 0.14	4.12 ± 0.93	< 0.001*	0.039*	< 0.001*
	C4/5	5.08 ± 1.47	6.08 ± 1.37	4.91 ± 1.11	0.329	0.756	0.038
	C5/6	3.63 ± 0.86	5.14 ± 1.16	0.69 ± 0.16	0.023*	< 0.001*	< 0.001*
	C6/7	2.76 ± 0.62	2.60 ± 0.59	2.83 ± 0.64	0.297	0.809	0.398
	Total	22.29 ± 5.03	17.70 ± 4.00	16.56 ± 3.74	0.027*	0.006*	0.498
Axial rotation							
	C2/3	2.15 ± 0.72	2.37 ± 0.55	1.97 ± 0.46	0.760	0.494	0.077
	C3/4	3.00 ± 0.69	0.53 ± 0.12	3.02 ± 0.70	< 0.001*	0.965	< 0.001*
	C4/5	3.90 ± 0.64	3.62 ± 0.84	3.69 ± 0.85	0.197	0.524	0.836
	C5/6	3.27 ± 0.69	3.29 ± 0.76	0.55 ± 0.13	0.642	< 0.001*	< 0.001*
	C6/7	2.24 ± 0.91	1.76 ± 0.41	1.59 ± 0.37	0.065	0.038*	0.303
	Total	14.56 ± 3.37	11.57 ± 2.67	10.82 ± 2.50	0.031*	0.007*	0.508

ROM, range of motion; Group 1, upper fusion and lower arthroplasty; Group 2, upper arthroplasty and lower fusion

*Statistically significant difference

### Intervertebral disc pressures

Table 4 shows the average pressures in intervertebral discs at different levels. The average intervertebral disc pressures of C2/3, C4/5, and C6/7 in Group 1 were significantly higher than that in the intact group in all directions (*P* < 0.005). In comparison between the intact group and Group 2, the average intervertebral disc pressures in Group 2 were significantly higher than that in the intact group (*P* < 0.005); however, no between-group difference was found at C2/3 in all directions (*P* > 0.005). The average intervertebral disc pressures at C2/3 were significantly higher in Group 1 than those in Group 2 in all directions (*P* < 0.005).

**Table 4:** Comparison of average pressures in intervertebral discs

Pressure (MPa)	Segments	Models			*P* values		
		Intact	Group 1	Group 2	Intact versus Group 1	Intact versus Group 2	Group 1 versus Group 2
Flexion							
	C2/3	0.25 ± 0.06	0.38 ± 0.10	0.30 ± 0.08	0.001*	0.078	0.038*
	C4/5	0.29 ± 0.06	0.49 ± 0.12	0.46 ± 0.12	< 0.001*	0.001*	0.586
	C6/7	0.26 ± 0.05	0.35 ± 0.09	0.49 ± 0.12	0.007*	< 0.001*	0.005*
Extension							
	C2/3	0.29 ± 0.06	0.40 ± 0.10	0.26 ± 0.07	0.005*	0.346	0.001*
	C4/5	0.29 ± 0.06	0.51 ± 0.13	0.49 ± 0.12	< 0.001*	< 0.001*	0.683
	C6/7	0.26 ± 0.05	0.34 ± 0.09	0.48 ± 0.12	0.020*	< 0.001*	0.005*
Lateral bending							
	C2/3	0.40 ± 0.07	0.64 ± 0.16	0.46 ± 0.12	< 0.001*	0.139	0.007*
	C4/5	0.43 ± 0.08	0.74 ± 0.19	0.74 ± 0.19	< 0.001*	< 0.001*	0.956
	C6/7	0.40 ± 0.07	0.53 ± 0.13	0.61 ± 0.15	0.001*	0.001*	0.260
Axial rotation							
	C2/3	0.43 ± 0.08	0.69 ± 0.17	0.50 ± 0.13	< 0.001*	0.165	0.006*
	C4/5	0.46 ± 0.08	0.78 ± 0.20	0.80 ± 0.20	< 0.001*	< 0.001*	0.884
	C6/7	0.43 ± 0.08	0.55 ± 0.14	0.66 ± 0.17	0.023*	< 0.001*	0.108

Group 1, upper fusion and lower arthroplasty; Group 2, upper arthroplasty and lower fusion

*Statistically significant difference

### Contact forces and pressures of facet joints

Table 5 shows the comparison of average contact forces in facet joints in extension between the intact group, Group 1, and Group 2. Compared with intact group, average contact forces in facet joints of C2/3 (*P* < 0.001), C4/5 (*P* < 0.001) and C6/7 (*P* < 0.001) were significantly higher in Group 1. Similarly, average contact forces in facet joints of C2/3 (*P* = 0.030), C4/5 (*P* = 0.005) and C6/7 (*P* < 0.001) were significantly higher in Group 2 than that in intact group. The average contact forces in facet joints of C2/3 (*P* = 0.007) were significantly more than that in Group 2; however, the average contact forces in facet joints of C6/7 (*P* < 0.001) in Group 1 were significantly less than that in Group 2 group. Table 6 shows the comparison of average contact pressures in facet joints in extension between the intact group, Group 1, and Group 2. Compared with intact group, average contact pressures in facet joints of C2/3 (*P* < 0.001) and C4/5 (*P* < 0.001) were significantly higher in Group 1 and Group 2 (*P* = 0.030 and *P* < 0.001, respectively); however, average contact pressures in facet joints of C6/7 (*P* < 0.001) were significantly less in Group 2 than those in Intact group. In comparison between Group 1 and Group 2, average contact pressures in facet joints of C2/3 (*P* = 0.007) were significantly less in Group 2, while average contact pressure in facet joints of C6/7 (*P* < 0.001) was significantly higher in Group 1.

**Table 5:** Comparison of average contact forces in facet joints in extension

Segments	Contact forces in models (N)			*P* values		
	Intact	Group 1	Group 2	Intact versus Group 1	Intact versus Group 2	Group 1 versus Group 2
C2/3	73.01 ± 9.51	98.17 ± 12.79	83.12 ± 10.83	< 0.001*	0.030*	0.007*
C4/5	81.69 ± 10.08	105.51 ± 13.74	105.49 ± 13.74	< 0.001*	0.005*	0.998
C6/7	80.11 ± 9.79	76.17 ± 9.92	104.83 ± 13.65	< 0.001*	< 0.001*	< 0.001*

Group 1, upper fusion and lower arthroplasty; Group 2, upper arthroplasty and lower fusion

*Statistically significant difference

**Table 6:** Comparison of average contact pressures in facet joints in extension

Segments	Contact pressures in models (Mpa)			*P* values		
	Intact	Group 1	Group 2	Intact versus Group 1	Intact versus Group 2	Group 1 versus Group 2
C2/3	1.87 ± 0.24	2.52 ± 0.33	2.13 ± 0.28	< 0.001*	0.030*	0.007*
C4/5	2.09 ± 0.26	2.71 ± 0.35	2.70 ± 0.35	< 0.001*	< 0.001*	0.998
C6/7	2.05 ± 0.25	1.95 ± 0.25	2.69 ± 0.35	0.360	< 0.001*	< 0.001*

Group 1, upper fusion and lower arthroplasty; Group 2, upper arthroplasty and lower fusion

*Statistically significant difference

## Discussion

The selection of surgical strategy in the treatment of noncontinuous cervical spondylopathy remains controversial, the difficulty of which lies in the preservation of intermedial intervertebral disc [14, 26]. It is reported that noncontinuous ACDF may theoretically bear additive stress from upper and lower fused levels with hypermobility in compensation, which will accelerate intervertebral disc degeneration [3, 29]. To decrease additive stress from fusion massed on the normal intermediate segment, long segmental anterior fusion is usually carried out in the treatment of noncontinuous cervical spondylopathy; long segmental anterior fusion always includes the intermediate segments [3]. However, this method is associated with a high risk of ASD and nonunion.

It is reported that noncontinuous TDR is a safe and effective treatment of noncontinuous cervical spondylopathy [8]. However, in multilevel TDR, the indication is more restricted, the technique is difficult, the prosthesis-related complication rate is high, and the cost is expensive [3, 30]. Our previous study showed that noncontinuous TDR required high biomechanical stability of the lower intervertebral level. This is because the stability of biomechanical structure and appropriate physiological curvature of the cervical spine could be required by noncontinuous TDR. Therefore, TDR is not suitable for patients with lower adjacent intervertebral disc degeneration [17]. HC contains the technical characteristics of ACDF and TDR. Therefore, it may be a good surgical strategy to solve the above problems.

The implementation of HC is still controversial. Most related studies apply HC in the treatment of C3 to C7 continuous cervical spondylopathy, which failed in the conservative treatment for more than six weeks [31]. In making optimal surgical strategy preoperatively, compensation of ROM, intervertebral stress, and facet joint force at adjacent levels should be taken into consideration [32].

Most previous studies used one sample, while anatomical differences could influence the ROM, intervertebral disc stress, and facet force [33, 34]. Therefore, multiple models were included in this study for statistical analysis to improve the accuracy of the conclusions. Our study showed that compared with intact FEM, HC mainly changed cervical movement characteristics in lateral bending and decreased the overall motion of the cervical spine. The possible reason is that ACDF changes the curvature of the cervical spine so that the cervical rotation centre shifts, and then the tension of cervical ligaments is increased by distraction [9, 35]. We found that the hypermobility of the intermedial intervertebral disc of Group 1 was more obvious than in Group 2, which implied that the protective effect of Group 1 on the intermedial intervertebral segment is weak.

In our study, average pressures in cervical intervertebral discs significantly increased after Group 1; however, Group 2 did not significantly change the physiological intervertebral disc pressure at the upper adjacent levels (C2/3). This suggests that Group 2 is closer to the physiological cervical biomechanical characteristics; Group 1 and Group 2 had similar effects on the average intervertebral disc pressure at the intermedial level (C4/5). Considering the mobility of the intermedial intervertebral disc of Group 1 was higher than Group 2, the protective effect of Group 1 on the intermedial intervertebral disc might be achieved by increasing segmental ROM to buffer the intervertebral disc pressure [24].

In this study, Group 1 could significantly reduce the contact forces in the facet joints of the lower adjacent segments, which implied Group 1 was suitable for patients with posterior column degeneration in lower adjacent segments. However, this was at the cost of increasing the contact forces of the upper adjacent facet joints. Both Group 1 and Group 2 could increase the contact forces in the intermedial facet joints. This indicates that neither surgical procedure is suitable for patients with severe posterior column degeneration at intermedial levels. In contrast, Wu et al. [26] reported that the biomechanical characteristics of the untreated segments were less influenced by HC. The reason might be that their study did not simulate the insertion procedure of implants; this would ignore the distraction of vertebrae after inserting implants. In addition, they discussed the HC, which included Zero-P and Prestige-LP. The design of Zero-P makes it difficult to improve cervical lordosis, especially in ACDF with multiple segments [36]. Prestige-LP can produce higher von Mises stress than Prodisc-C at the posterior flanges on the end plate [21]. Therefore, the satisfactory biomechanical characteristics obtained in the treatment of noncontinuous cervical spondylopathy with the above-mentioned HC are at the expense of increasing the internal stress of implants. These two implants may not be recommended for this type of procedure. There are still some limitations in this study, which require further improvement in future work. First, although image data of 12 volunteers are included in this study for modelling, it is difficult to completely simulate the changes in biomechanical characteristics of intervertebral disc degeneration. Second, the depth of implants has a great impact on the cervical biomechanical properties after HC. In this study, only standardized implant depth can be used for analysis; it is difficult to carry out an in-depth discussion on a more complex situation. Therefore, we can only use FEA to study the trend of biomechanical characteristics of the FEMs. In future studies, we will use artificial intelligence technology-assisted FEA to improve research methods, hoping to further improve the accuracy.

## Conclusions

The results of our study show that HC has high requirements on biomechanical characteristics of unoperated cervical vertebra segments in the treatment of noncontinuous cervical spondylopathy. The protective effect of fusion-arthroplasty on the intermedial intervertebral segments is weak. Arthroplasty-fusion is closer to the physiological cervical biomechanical characteristics in the upper adjacent segments. Therefore, Arthroplasty-fusion is preferred for intervertebral disc degeneration in adjacent upper segments. Fusion-arthroplasty can significantly reduce the average pressures in intervertebral discs and contact forces of facet joints at lower adjacent segments. Therefore, fusion-arthroplasty is preferred for the treatment of patients with lower intervertebral disc degeneration or lower posterior column degeneration.

## Data Availability

Please contact author for data requests.

## Abbreviations

ACDF: Anterior cervical discectomy and fusion
ASD: Adjacent segment degeneration
TDR: Total disc replacement
HC: Hybrid construction
ROM: Range of motion
FEA: Finite element analysis
CT: Computed tomography
FEM: Finite element model
ALL: Anterior longitudinal ligament
PLL: Posterior longitudinal ligament
LF: Ligament flavum
IL: Interspinous ligament
SL: Supraspinous ligament
CL: Capsular ligament
SD: Mean ± standard deviation
